# Unsupervised statistical clustering of environmental shotgun sequences

**DOI:** 10.1186/1471-2105-10-316

**Published:** 2009-10-02

**Authors:** Andrey Kislyuk, Srijak Bhatnagar, Jonathan Dushoff, Joshua S Weitz

**Affiliations:** 1 School of Biology, Georgia Institute of Technology, Atlanta, GA 30332, USA; 2 UC Davis Genome Center, University of California, Davis, Davis, CA 95616, USA; 3 Department of Biology, McMaster University, Hamilton, Ontario L8S 4K1, Canada; 4 School of Physics, Georgia Institute of Technology, Atlanta, GA 30332, USA

## Abstract

**Background:**

The development of effective environmental shotgun sequence binning methods remains an ongoing challenge in algorithmic analysis of metagenomic data. While previous methods have focused primarily on supervised learning involving extrinsic data, a first-principles statistical model combined with a self-training fitting method has not yet been developed.

**Results:**

We derive an unsupervised, maximum-likelihood formalism for clustering short sequences by their taxonomic origin on the basis of their *k*-mer distributions. The formalism is implemented using a Markov Chain Monte Carlo approach in a *k*-mer feature space. We introduce a space transformation that reduces the dimensionality of the feature space and a genomic fragment divergence measure that strongly correlates with the method's performance. Pairwise analysis of over 1000 completely sequenced genomes reveals that the vast majority of genomes have sufficient genomic fragment divergence to be amenable for binning using the present formalism. Using a high-performance implementation, the binner is able to classify fragments as short as 400 nt with accuracy over 90% in simulations of low-complexity communities of 2 to 10 species, given sufficient genomic fragment divergence. The method is available as an open source package called LikelyBin.

**Conclusion:**

An unsupervised binning method based on statistical signatures of short environmental sequences is a viable stand-alone binning method for low complexity samples. For medium and high complexity samples, we discuss the possibility of combining the current method with other methods as part of an iterative process to enhance the resolving power of sorting reads into taxonomic and/or functional bins.

## Background

Metagenomics, the study of the combined genomes of communities of organisms, is a rapidly expanding area of genome research. The field is driven by environmental shotgun sequencing (ESS), a technique of applying high-throughput genome sequencing to non-clonal DNA purified directly from an environmental sample. This removes the requirement to isolate and cultivate clonal cultures of each species, allowing an unprecedented broad view of microbial communities.

Thus far, environments such as acid mine drainage [1], Scottish soil [2], open ocean [3], termite gut [4], human gut [5], and neanderthal [6] have been sequenced, to name a few. Attention has been directed to bacterial and viral fractions of these communities, with eukaryotic metagenomics pioneered by projects such as the marine protist census [7]. Complexity of these communities varies greatly from 5 to several thousand identifiable bacterial species. These projects have uncovered vast amounts of previously unobserved genetic diversity [8,9]. For example, "deep sequencing" using 454 pyrosequencing suggests that possibly tens of thousands of species coexist in a single ml of seawater [10].

Given this wealth of genomic data it is becoming possible to make increasingly precise biological inferences regarding the structure and functioning of microbial communities [11-13]. As but one example, the discovery of a novel proteorhodopsin gene was the first step in uncovering a previously unknown, yet apparently dominant, mechanism for phototrophy in the oceans [14]. Characterization of functional diversity is limited by our ability to classify sequences into distinct groups that reflect a desired taxonomic or functional resolution.

Shotgun metagenomic DNA is sequenced in fragments of 50 to 1000 nucleotides, then possibly assembled into longer sequences (contigs). Phylogenetic binning, the task of classifying these sequences into bins by taxonomic origin, then becomes critical to separate metagenomic data into coherent subsets plausibly belonging to separate organisms. This task is challenging due to the short length of available fragments. Bacterial communities of very high complexity, with thousands of species present, further complicate the task.

While methods such as 16S bacterial community censuses [15] and functional- or sequence-based screening surveys are the forerunners of modern metagenomics, indiscriminate whole-genome shotgun sequencing may be the defining approach of the discipline today. This approach has recently generated vast amounts of data, facilitated by continual capacity increases and quality improvements at major sequencing centers and the emergence of cost effective very high throughput Next Generation sequencing (NGS) (454 pyrosequencing [16], Illumina [17] and SOLiD [18]). At the highest diversity levels, the reads may not be assembled at all due to the sparseness of even the highest throughput sequencing methods and the danger of chimeric assemblies, arising from sampling so many organisms at once, leaving the binner with raw reads. Binning methods therefore aim to be able to operate on very short read lengths provided by next-generation sequencing, although most, including the present approach, are only able to go down to 454 pyrosequencing read length (about 400 nt) and not to microread length (30 to 100 nt).

Classic approaches to phylogenetic determination of species identities from environmental sequences rely on identifying variants of highly conserved genes, like 16S rRNA or recA [19]. This approach is not applicable on a full metagenomic scale for two reasons: first, ribosomal or marker gene sequences comprise a small fraction of the bacterial genome, so most shotgun sequences do not contain them and cannot be classified this way; and second, organisms with identical or closely related 16S genes have been shown to exhibit variations in essential physiological functions [20]. Other approaches are broadly divided into sequence similarity based classifiers such as MEGAN [21], which rely on BLAST or other alignments, and sequence composition based classifiers, which rely on statistical patterns of oligonucleotide distributions. Many solutions integrate the task of phylogenetic assignment (labeling) together with that of binning per se (clustering) of genomic fragments. However, with unsupervised methods, like the one presented here, labeling is not possible as part of the algorithm and has to be performed by other means, like analyzing the correspondence of generated clusters to known phylogenies.

Sequence classification based on oligonucleotide distributions has been the basis for gene finding applications since the early 1990s. In 1995, Karlin and Burge [22] noted that dinucleotide distribution is relatively constant within genomes but varies between genomes. Since then, this property has been extensively studied and generalized to other oligonucleotide lengths [23]. With the advent of ESS, several binning methods have used oligonucleotide distributions of various orders to build supervised and semi-supervised classifiers. These include PhyloPythia [24], CompostBin [25], and self-organizing map (SOM) based methods [26-28].

Machine learning-based classification algorithms like those used for binning are categorized into supervised, semi-supervised, and unsupervised classes. Supervised algorithms accept a training set of labeled data used to build their models, which are then applied to the query data. In case of binning, this training set consists of genomic sequences labeled according to the species they originate from. Semi-supervised algorithms use both training set data and query data to build their models. Unsupervised algorithms use no training data and derive their models directly from the query input. While methods described above have achieved considerable success in classifying short anonymous genomic fragments, their supervised nature makes them reliant on previously sequenced data. For example, BLAST-based methods are completely dependent on the presence of sequences related to the query in the database. While semi-supervised clustering methods can have significant generalizing power, their accuracy still depends on similarity of input data to their training set.

To our knowledge, two approaches to unsupervised metagenomic binning have been published. TETRA [29,30] explores the applications of *k*-mer frequency statistics to metagenomic data. The authors state that their method is suitable as a "fingerprinting technique" for longer DNA fragments, though not as a general-purpose binning method for single-read 454 pyrosequenced or Sanger fragments, and an application of methods including TETRA to binning of fosmid-sized DNA is used in [31]. Abe *et al*. [26] used self-organizing maps (SOM) in combination with principal component analysis (PCA) on 1- and 10-Kb fragments, and this method was evaluated and enhanced in [27] using growing self-organizing maps (GSOM), an extension of SOM, on 8- and 10-kb fragments.

Given the apparent diversity of metagenomic samples and the significant fraction of the full bacterial phylogeny with no sequenced representatives [20,32], as well as possible undiscovered diversity of the tree of life, binning methods must perform well on previously unseen data. Semi-supervised methods may be able to extrapolate on this data, but if not, unsupervised clustering will be a necessary part of a combined-method binning approach. We present LikelyBin, a new statistical approach to unsupervised classification of metagenomic reads based on an explicit likelihood model of short genomic fragments [33]. The rest of this paper is organized as follows. The Methods section introduces a formal definition of the binning problem, the application of the Markov Chain Monte Carlo (MCMC) formalism, and the feature space and likelihood model used. We discuss numerical methods used in the implementation, including a novel coordinate transformation which achieves dimension reduction for the feature space of *k*-mer frequencies, and the genomic fragment divergence measure *D*_*n*_, a novel statistical measure we developed for performance evaluation of our algorithm. The Results section presents performance evaluations of our method on mixtures of 2 to 10 species compiled from completed genomes available in GenBank, with fragment lengths starting at 400 nt, as well as accuracy trends over different fragment lengths and mixing ratios. We also present results on the FAMeS [34] dataset and compare the current method to a semi-supervised binning method based on *k*-mer distributions [25]. The Conclusion section explains the applicability of our method, its speed and availability, as well as important future directions for improvement.

## Methods

### The binning problem

We state the problem as follows: given a collection of *N *short sequence reads from *M *complete genomes, how can we predict which sequences derive from the same genome? In our model, we represent a genome as a string of characters deriving from a stochastic model with parameters Θ, referred to here as a master distribution. We make the simplifying assumption that the oligonucleotide distribution is uniform across the bacterial chromosome. This assumption is not satisfied biologically; gene-coding, RNA-coding, and noncoding regions, leading and lagging strands of replication, and genomic islands resulting from horizontal gene transfer can all exhibit distinct oligonucleotide distributions. Accurate classification of these regions in metagenomic fragments is an open problem which requires complex statistical models that we have yet to incorporate into our framework, and which are targets for subsequent model development. Nonetheless we have found that clustering of short reads using the above assumption is sufficiently accurate for use in low complexity metagenome samples.

Given this assumption of statistical homogeneity, we model a collection of sequences from a single genome as realizations of a single stochastic process. Similarly, we model a collection of sequences from multiple genomes as realizations of multiple stochastic processes, one per genome, each with its own master distribution. We are interested in determining which sequences in a metagenomic survey are likely to have been drawn from the same genome and, consequently, the statistical distributions of oligonucleotides within each of the master distributions. If the number of master distributions is unknown, then we must include some prior estimate to close the model. Thus, even in cases where due to insufficient coverage it is impossible to assemble disparate segments of a consensus genome together, a binning algorithm should still be able to group reads together based on their statistical distribution of oligonucleotides.

The simplest model of a genome would be a random collection of letters, A, T, C, and G. The master distribution of a single genome can then be represented as a single probability, *p*_*A*_, denoting the fraction of A-s in the genome. Base complementarity requires *p*_*A *_= *p*_*T *_and *p*_*C *_= 1/2 - *p*_*A *_= *p*_*G*_. A more complex representation would be to assume that genomes are random collections of *k*-mers. When *k *= 1, each nucleotide is independent of the previous. When *k *= 2, the genomes are random collections of dimers and so on. However, when *k *≥ 2, inherent symmetries are present in this representation since all but the first letters of the current *k*-mer are also contained in the next *k*-mer. In a metagenomic dataset, each short fragment derives from a single master distribution, *θ*_*i*_, which is represented a fraction *f*_*i *_of times. How then can we infer the most likely Θ ≡ (*θ*_1_, *θ*_2_, ..., *θ*_*M*_) and *F *≡ (*f*_1_, *f*_2_, ..., *f*_*M*_) given a set of *N *sequences *S *≡ (*s*_1_, *s*_2_, ..., *s*_*N*_)? To do so, we must calculate the likelihood ℒ(*S*|Θ, *F*) of observing the sequences *S *given the parameters Θ and *F*. Then, we must estimate the values of Θ and *F *that maximize the likelihood ℒ. Below, we demonstrate the use of a MCMC algorithm to perform this task.

### MCMC framework

**Figure 1:**
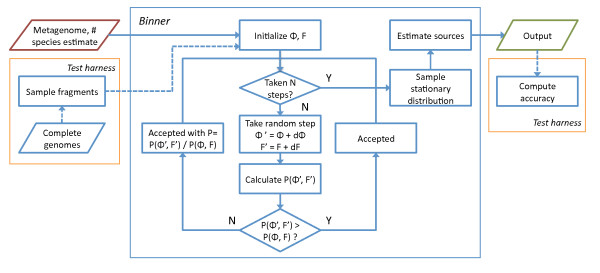
**Binning diagram**. Diagram of binning data pathways and main MCMC iteration loop.

FIGURE 1 We are interested in finding the values of Θ and *F *that maximize the likelihood, ℒ. The MCMC approach has been described in detail elsewhere [35]. Given an initial parameter setting and a metagenomic data set, we implement the following Metropolis-Hastings algorithm to MCMC maximum likelihood estimation: (i) Determine the likelihood of the dataset ℒ(Θ, *F*|*S*); (ii) Choose some Φ = Θ + dΘ, and *G *= *F *+ dF and determine its likelihood, ℒ*' *(Φ, *G*), such that both Φ and *G *exist in the same high-dimensional simplex as Θ and *F *respectively; (iii) Accept the new value given a probability 1 if ℒ*' *(Φ, *G*) > ℒ'(Θ, *F*) and with probability ℒ*' *(Φ, *G*)/ℒ(Θ, *F*) otherwise; (iv) Repeat, and after a burn-in period determine the values  and  which maximize ℒ(*S*|Θ, *F*). We can then utilize the resulting model of sequence parameters to classify sequences and estimate the most likely oligonucleotide distribution of each of the originating master distributions. The iterative process, together with key stages of the entire binning algorithm, is illustrated in Figure 1. Some technical details necessary for the implementation follow.

#### Likelihood model

Consider a nucleotide sequence *s *= *c*_1 _*c*_2 _*c*_3_, ..., *c*_ℓ_. We would like to know the probability of observing such a sequence given some underlying model. We assume that our sequence is selected from broken pieces of double-stranded DNA, and thus that complementary nucleotide sequences have the same probability: i.e., *L*(*s*) = *L*(*s'*), where , and  is the nucleotide complementary to the nucleotide *c*_*i*_. We assume that the probability of our sequence is determined by a set of 2^*k *^*k*-mer probabilities .

That is, we write:

(1)

Assuming we know probabilities for all of our *k*-mers, we have probabilities for *k *- 1-mers as marginals.

Thus we can write:

(2)

As an example, the probability of a sequence given a set of known dimer frequencies is:

(3)

Note that we assume the marginal probabilities are well defined: i.e., that we get the same marginal probability if we collapse a *k*-mer to a *k *- 1-mer by summing over the first, or the last, nucleotide. The likelihood of observing *N *sequences given *M *master distributions is

(4)

where *P*_*m*_(*s*_*i*_) is the probability of generating the *i*-th sequence given the *m*-th master distribution.

A simple example of likelihood computation according to the described model is given in the Appendix.

#### The space of *k*-mer frequencies

Given the assumption of uniformity of the *k*-mer (oligonucleotide) distribution across each genome, we can impose three kinds of constraints on the *k*-mer frequency space. This space is a subspace of , subject to three kinds of constraints: all *k*-mer frequencies sum to 1, e.g.



each *k*-mer has the same frequency as its complement; and all marginal probabilities are consistent over all margins, e.g.



We then derive a transformation of the original *k*-mer frequency vector, *x *= [*p*_*A*_, *p*_*T*_, *p*_*G*_, *p*_*C*_, *p*_*AA*_, *p*_*AT*_, *p*_*AG*_, *p*_*AC*_, *p*_*TA*_, ...], into the independent coordinate space. To generalize and automate the process, we perform it for each case from 1-mers (4 dimensions before removing redundancies) to 5-mers (1364 dimensions before removing redundancies) by generating all equations governing the constraints above. We use the notation [*A*|*b*] to denote the matrices of the constraint equation *Ax *= *b *by generating rows for each constraint type. For example, for *k *= 2, we write the summation, complementarity and marginality constraints as follows:

(5)

(6)

(7)

We find the nullspace of the resulting matrix *A *and use it to perform the transformation. The resulting number of independent dimensions is shown in Table 1. The MCMC simulation then performs the search in the independent coordinate space. For *k *> 6, the matrix *A *becomes too big to compute its nullspace using a non-parallelized algorithm. Even for *k *= 6, the number of independent dimensions is so large that the MCMC simulation takes an intractable amount of time. Therefore, we only generalize our algorithm up to *k *= 5.

**Table 1:** Redundancies in oligonucleotide dimension space

***k***	**Total dimensions**	**Independent dimensions**
1	4	1

2	20	7

3	84	25

4	340	103

5	1364	391

#### Initial conditions

The choice of initial conditions can dramatically alter the speed of convergence of a MCMC solver. We used the same initial conditions for comparison of model results, specified by the frequencies of *k*-mers in the entire dataset provided as input (i.e., the weighted average of all sources' contributions to the dataset). Other possibilities, implemented but not chosen as the default, include taking uniformly distributed frequencies, randomizing the starting condition, or using principal components analysis with *K*-means clustering to obtain initial cluster centroids. We verified that convergence, when it did occur, did not depend sensitively on initial conditions (Additional files 1 and 2).

#### Finding the maximum likelihood model

Once the predefined number of timesteps has elapsed, the model with the largest log likelihood is selected. Note that the MCMC framework is amenable to a Bayesian approach, which we implemented as an alternative. Once the equilibrium state has been reached we calculate the autocorrelation of frequencies and estimate a window over which frequencies show no significant autocorrelations. Given a specified prior distribution *p*(Θ, *F*) for the master distribution and frequencies, the Metropolis-Hastings approach will converge to the true posterior distribution of π (Θ, *F*|*S*) ∝ ℒ (*S*|Θ, *F*) *p*(Θ, *F*). In our case we used an uninformed prior distribution so long as positivity and all other specified constraints among *k*-mer probabilities were preserved. We then sample from the equilibrium state to find π (Θ, *F*). Averages of master distributions in the posterior distribution also preserve the constraint conditions because of the linearity of the averaging operator. Accuracy of the model was similar whether using the maximum likelihood model or the average of the posterior distribution (Additional file 3). Full posterior distributions of *k*-mer models could be used to estimate posterior distributions of binning accuracy.

## Numerical details

### Precision

Due to precision limitations of the machine double precision floating point format, the model likelihood calculation is performed in log space. Denote the old model under consideration as **M **= {*M*_1_, *M*_2_, ... *M*_*m*_}, and the new (perturbed) model as . The log likelihood of a single model is



and note that the innermost fraction contains higher-order terms when working with Markov chain orders higher than 2. The innermost product term is a product of on the order of 1000 terms of magnitude ≈ 1/4. However, 1/4^*n *^exceeds double floating point precision at *n *≈ 540. To prevent underflow, we find the *P*_*m*_(*s*_*i*_) of highest magnitude and divide the inner sum by it. This allows log space evaluation of the highest magnitude term and ensures that any terms whose precision is lost are at least ≈ 1e300 times smaller. The model log likelihood ratio is then . If this term exceeds 0, the new model is more likely to be observed than the old.

The MCMC iteration loop was implemented with the Metropolis-Hastings criterion. From an initial model, a perturbed model *M*_*N *_is generated. The new model's probability is evaluated as above and compared to that of the currently selected model *M*_*C*_. If higher, the new model is selected; otherwise, the new model is selected with probability *p *= exp (log ℒ(*M*_*N*_|*S*) - log ℒ(*M*_*C*_|*S*)). The step is repeated *N *times (*N *is fixed at 40000 for the experiments described). Each selected model is stored in a model record for later sampling.

#### Computing the perturbation

The statistical model consists of sub-models for each source. The perturbation step is performed for every sub-model independently. Every sub-model consists of a complete *k*-mer frequency vector, {*p*_*A*_, *p*_*T*_, *p*_*G*_, *p*_*C*_, *p*_*AA*_...}. It is perturbed by scaling each vector of the basis matrix *A *by a random number *r*_*i *_drawn from a Gaussian distribution with mean 0 and constant variance (computed as described below), then adding each scaled vector in succession to the frequency vector. The basis matrix *A *is precomputed for each *k*-mer model order from 2 to 5 and supplied with the program. The computation is performed by generating a system of equations representing the base complementarity, marginal, and summation constraints and using the standard nullspace algorithm supplied with GNU Octave.

The perturbation step variance must be calibrated independently for each dataset. An excessive variance will result in too many suboptimal perturbations as well as perturbations placing the frequency vector outside the unit hypercube (those perturbations are rejected). A variance that is too small can result in an inability to escape local maxima in the model search space and an inability to reach the stationary phase before the pre-determined number of steps is taken. To calibrate the variance, the MCMC iteration is started independently for a reduced number of steps, and different variances ranging from 1*e *- 3 down to 1*e *- 8 are tried. With each trial, the number of new model acceptances is recorded. We consider the fraction . Once the variance yielding *f *closest to 0.234 is found (a heuristic level of acceptances that has become standard [35], p. 504), we use this variance for the main run. Convergence to the stationary phase occurred after 40,000 iterations in all cases of interest.

#### Computing the prediction

To derive the final model prediction, the model with the overall maximum log likelihood is selected. The full MCMC simulation is repeated a selected number of times (to increase performance, the classifier was run in parallel on an 8-core machine; each core was assigned to run one MCMC simulation for a total of 8 restarts). Final model predictions are compared between different runs, and the best overall prediction is selected according to its model likelihood (described above).

**Figure 2:**
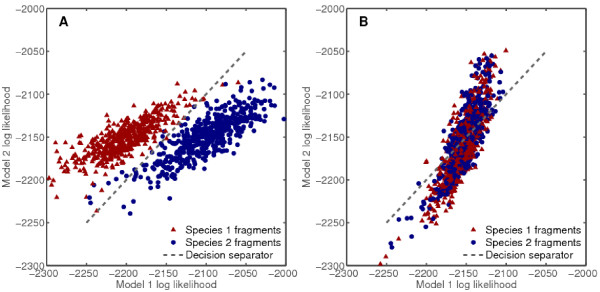
**Fragment likelihood separation**. Log likelihood values of fragments from pairs of species according to models fitted by the classifier. Points' positions on the two axes represent log likelihoods of each fragment according to the first and second model, respectively. A, *Helicobacter acinonychis *vs. *Vibrio fischeri*, good separation (98% accuracy, D = 1.31); B, *Streptococcus pneumoniae *vs. *Streptococcus pyogenes*, poor separation (57% accuracy, D = 0.22). Fragment length was 800 in both cases. 500 fragments per species were supplied.

The classifier then assigns a putative source to each sequence fragment it was initially queried with. For every fragment, its likelihood according to each sub-model in the final predicted model is computed, and the sub-model supplying the highest likelihood is selected. Since the sources are anonymous, they are referred to simply by indices from 1 to *n *corresponding to each sub-model's index in the final predicted model. Figure 2 illustrates the log likelihood comparison process for all fragments in a given dataset, according to the best model selected as a result of this process.

### Testing methodology

Simulated metagenomic datasets were created by selecting two or more genomic sequences as source DNA. Sequence fragments were selected at random positions within source sequences; overlaps were allowed to occur. Fragment size was fixed for all fragments for each experiment. The total number of fragments per source was selected either according to overall source length or at specified frequency ratios (e.g., 2:1, 10:1:1). The number of sources in each testing dataset was supplied to the classifier.

Accuracy of the classifier is calculated as follows. Every possible matching of source genomic sequence names to classifier output indices is considered, e.g. {*seq*1 → 1, *seq*2 → 2}, {*seq*1 → 2, *seq*2 → 1}. The number of correct assignments made by the classifier is then counted for each matching and the matching with the highest number of correct assignments is selected. Accuracy is then given as . To evaluate separability of the randomly generated datasets according to the classifier's model, we also define and compute the *genomic fragment divergence *between two sources' *k*-mer distributions. First, we compute the mean, *μ*, and standard deviation, *σ*, of each *k*-mer frequency for each source across fragments originating from that source. The genomic fragment divergence of *k*-mer order *n *is then given by

(8)

Generalizing to *M *species, let {*S*} = {*S*_1_, *S*_2_, ... *S*_*m*_}. Then we define.

(9)

**Figure 3:**
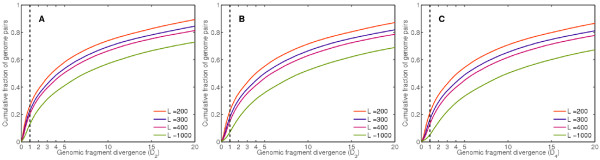
**Pairwise genome divergence distributions**. Cumulative distributions of pairwise divergences (*D*_*n*_) between all completed bacterial genomes retrieved from GenBank. Fragment lengths of 400 to 1000 were used to compute *D*_*n*_. Divergences based on *k*-mer order 2, 3, and 4 are represented in panels A, B, and C, respectively. The vertical cut-off line at *D *= 1 indicates an empirical boundary above which the binning algorithm works with high accuracy. For fragment length 400, over 80% of all randomly selected pairs are observed to have divergences above this line.

Figure 3 illustrates the distribution of genomic fragment divergences between completed bacterial genomes. A different formula for intergenomic difference, called the *average absolute dinucleotide relative abundance difference *is [36]: , where . This formula encompasses dinucleotides and pairwise comparisons of entire sequences only, and uses dimer frequency biases instead of absolute frequencies and their deviations in a hierarchical fashion.

## Results and Discussion

The accuracy and applicability of the present method in binning short sequence fragments from low complexity communities (2-10 species) was systematically analyzed using a variety of species, varying fragment lengths, and varying ratios of fragment representation.

**Figure 4:**
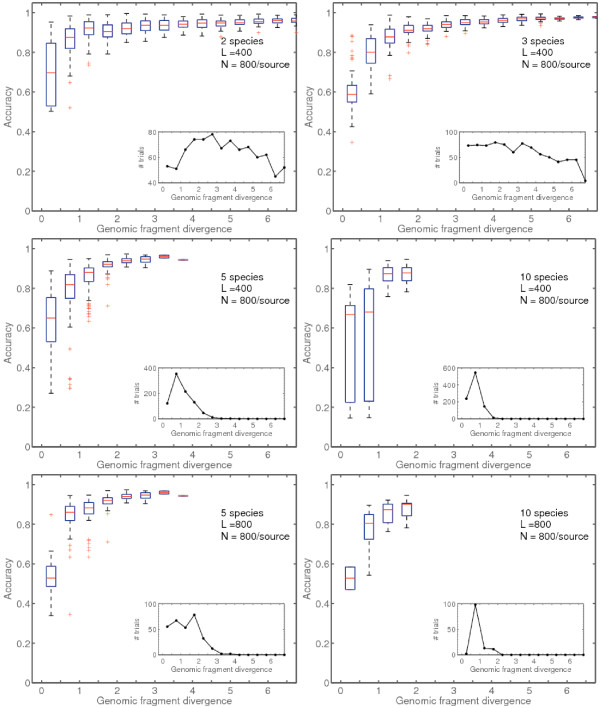
**Algorithm accuracy vs. fragment divergence**. Sets of 2, 3, 5, 10 genomes were sampled randomly from a set of 1055 completed bacterial chromosomes, and experiments were conducted as described in Materials and Methods. Trials were conducted with 400- and 800-nt long fragments. Classification accuracy for the majority of genome pairs above overall divergence 1 is in the high performance range (accuracy > 0.9), while above divergence 3 accuracy is above 0.9 for over 95% of the trials. Results for Bayesian posterior distribution sampling were not significantly different (Additional file 3).

First, a set of 1055 completed bacterial chromosomes was retrieved from GenBank. This set was randomly sampled for sets of 2, 3, 5, 10 genomes at a time, representative of various genomic fragment *k*-mer distribution divergences. Binning results for nearly 1800 simulated communities comprised of 2 or 3 genomes at a time are summarized in the top panels of Figure 4. There is a strong positive correlation between genomic fragment divergence and average performance. Classification accuracy was consistently above 85% for fragment divergences when *D*_3 _> 2. Results for Bayesian posterior distribution sampling were not substantially different (Additional file 3).

Accuracy of binning simulated communities of 5-10 species was consistent with the results from 2-3 species communities. The accuracy of binning was strongly positively correlated with genomic fragment divergence with accuracies consistently above 85% for *D*_3 _> 2. Note that accurate binning was possible when fragment length was either *L *= 400 nt or *L *= 800 nt (middle and bottom panels of Figure 4 respectively). For 5 and 10 species, a total of 1815 simulated communities were tested in the *L *= 400 nt case and a total of 425 simulated communities were tested in the *L *= 800 nt case.

**Figure 5:**
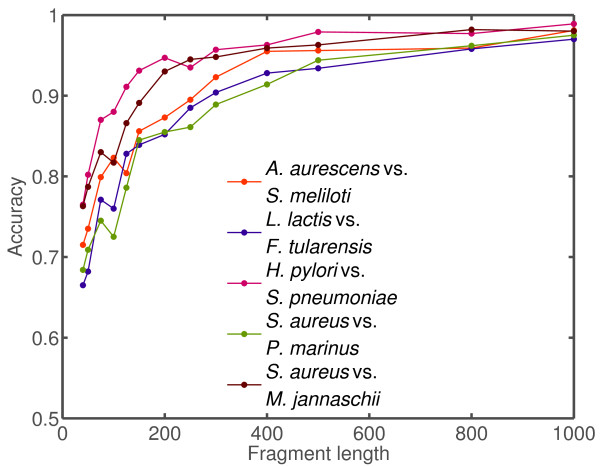
**Algorithm accuracy vs. fragment length**. Fragment length-dependent performance on 2-species datasets. Same trials as in Figure 4 were performed on a subset of pairs of genomes while varying simulated fragment size from 40 to 1000. The species' characteristics are given in Table 2.

Next, we evaluated the robustness of our binning method to changes in fragment length and to changes in fragment ratios using five distinct genome pairs from the preceding experiment (see Table 2). The pairs were selected based on their relatively low genomic fragment divergence, *D*_3 _≈ 1, given a fragment length of *L *= 400 nt. Binning results on these 2-species tests were evaluated using sequence fragments whose lengths ranged from 40 to 1000 nt. The results are shown in Figure 5. Performance stabilizes close to its optimal value at fragment length 400. Again, results for Bayesian posterior distribution sampling were not substantially different than the maximum likelihood approach (Table 3).

**Table 2:** Summary of species' characteristics, including all independent monomer and dimer frequencies, in the subset of trials on 5 pairs of genomes performed in Figures 5 and 6.

**Species composition**	**GC content**	***p*_*A*_**	***p*_*AA*_**	***p*_*AC*_**	***p*_*AT*_**	***p*_*CA*_**	***p*_*CG*_**	***p*_*GC*_**
*Arthrobacter aurescens TC1*	63%	0.186	0.041	0.044	0.048	0.054	0.127	0.114
*Sinorhizobium meliloti 1021*	62%	0.189	0.040	0.057	0.037	0.068	0.097	0.098

*Lactococcus lactis subsp. cremoris MG1363*	36%	0.322	0.128	0.046	0.092	0.063	0.025	0.037
*Francisella tularensis subsp. holarctica FTA*	32%	0.337	0.118	0.047	0.109	0.059	0.015	0.038

*Helicobacter pylori HPAG1*	40%	0.301	0.105	0.050	0.082	0.066	0.027	0.042
*Streptococcus pneumoniae R6*	39%	0.303	0.126	0.040	0.079	0.058	0.037	0.060

*Staphylococcus aureus RF122*	35%	0.324	0.122	0.042	0.097	0.060	0.017	0.037
*Prochlorococcus marinus str. NATL2A*	33%	0.333	0.121	0.053	0.110	0.066	0.026	0.035

*Staphylococcus aureus subsp. aureus COL*	31%	0.343	0.134	0.038	0.110	0.055	0.008	0.027
*Methanocaldococcus jannaschii DSM 2661*	33%	0.335	0.122	0.053	0.112	0.065	0.026	0.033

**Table 3:** Summary of algorithm performance on JGI FAMeS data.

**FAMeS identifiers**	**min *D*_3_**	**Fragment count**	**Fragment length**	**Accuracy**
APOW1005, PPD1199, AIBF1022, AHZI1134, AHXO1014	2.3451	500	400	0.87

BCSB1222, ABFI1048, AHYP1295, AKNK1296, AAZH3626	1.9598	500	400	0.69

AHYT1136, AHYI1010, PIT10099, AINZ1029, AHZF1044	1.9314	500	400	0.85

PPD1199, AUNI1013, ABSU1031, AABS2846, AHXO1014	1.8881	500	400	0.89

AOTU1003, BCSB1222, AIOH1083, AIFS1040, AHXX1063	1.8032	500	400	0.86

BCSB1222, VNY1182, AHXF1121, AKNK1296, AHZI1134	1.3563	500	400	0.81

KPY1561, AOTY1222, BAHF1005, POG1025, AAOP1172	1.2429	500	400	0.79

BCSB1222, AADD1003, AUNI1013, KPR1102, AHXO1014	1.1571	500	400	0.87

AICI1287, AAOO1711, AKNK1296, AHXX1063, KPR1102	1.0279	500	400	0.72

AHYT1136, AAWX1070, WBJ1361, AIAI1092, AXBY1147	0.9987	500	400	0.65

AICI1287, AHYT1136, AAWX1070, AADE1259, AINZ1029	0.9856	500	400	0.72

AUSC1572, AHYF1232, AAON1449, AIAX1019, ACBK1133	0.8884	500	400	0.78

Average (12 trials, 5 sources, *L *= 400)	1.46	500	400	0.79

Random subsets of 5 sources each were selected from the FAMeS simLC dataset, with a genomic fragment divergence, *D*_3_, as shown. Fragments were truncated to the indicated length where appropriate. Reads from the dataset were used raw with no trimming.

**Figure 6:**
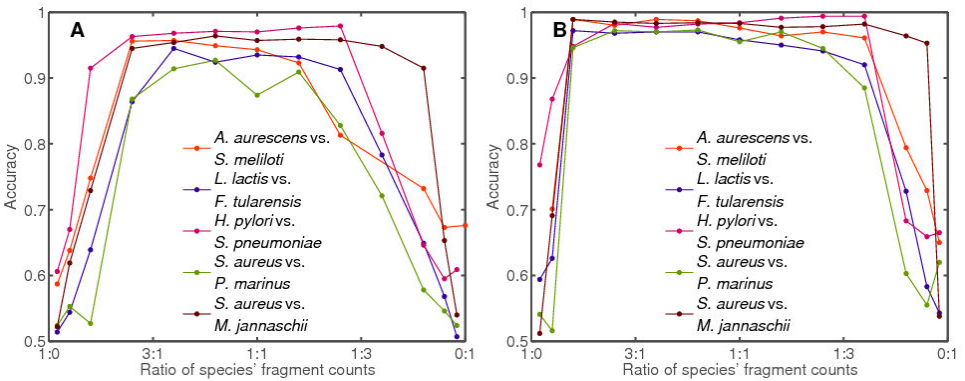
**Algorithm accuracy vs. source ratio**. Fragment ratio-dependent performance on 2-species datasets. Same trials as in Figure 4 were performed on a subset of pairs of genomes while varying species' contributions to the dataset from 2% to 98%. Fragment sizes were fixed at 400 nt (A) and 1000 nt (B). The species' characteristics are given in Table 2.

For the same five pairs as in Figure 5, we performed a test of fragment ratio-dependent contributions to accuracy (Figure 6). The binner successfully classifies mixtures with species' fractional content of 20% and above. Although robust to moderate variation in fragment ratios, these results indicate that binning relatively rare species may require modifications to the present likelihood formalism.

We also tested our method using subsets of the JGI FAMeS [34,37] simulated low-complexity dataset (simLC). We took 5 genomic sources at a time, using 500 fragments, each of length *L *= 400 nt. The accuracy results for binning these simulated low complexity communities are summarized in Table 4. The binning method has approximately 80% accuracy for a five-species community despite the genomic divergence, *D*_3_, being approximately 1.5 (an indicator of a community with similar *k*-mer distributions).

**Table 4:** Performance comparison of LikelyBin and CompostBin on pairs of genomes analyzed in Figures 5, 6, Table 2.

**Org 1**	**Org 2**	**Frag L**	**Frag N**	***D*_3_**	**LikelyBin accuracy**	**CB seeds**	**CompostBin accuracy**
*S. meliloti*	*A. aurescens*	400	500	1.02	0.94	10 25	0.93 0.93

*L. lactis*	*F. tularensis*	400	500	1.15	0.92	10 25	0.76 0.12*

*S. pneumoniae*	*H. pylori*	400	500	0.97	0.96	10 25	0.12* 0.96

*P. marinus*	*S. aureus*	400	500	0.99	0.93	10 25	0.73 0.83

*M. jannaschii*	*S. aureus*	400	500	0.92	0.94	10 25	0.17* 0.91

*Frag L*, Fragment length; *Frag N*, Number of fragments per source; *CB seeds*, labeled fragments supplied to CompostBin for training. LikelyBin consistently performed equally to or above CompostBin performance despite being completely unsupervised, while CompostBin required a fraction of input fragments to be labeled to seed its clustering alorithm. We supplied training fragments to CompostBin without regard to their origin (protein or RNA-coding). In a likely practical scenario, only 16S RNA-coding fragments would be labeled, but would have different *k*-mer distributions from protein-coding regions, possibly confounding classification. (*) Convergence toward a good clustering was not observed in CompostBin for these datasets; accuracy can be less than 50% due to labeled input.

We also compared our method to CompostBin [25], a semi-supervised algorithm that utilizes a PCA method to bin fragments based on their *k*-mer distributions (Table 5). We performed comparisons on pairs of genomes with fragment divergence *D*_3 _≈ 1 using the same dataset analyzed in Figures 5, 6 and Table 2. The results indicated that our method performs on par with or better than CompostBin, even though CompostBin required a fraction of input fragments to be labeled to initialize its clustering algorithm. Run time and memory performance was comparable between the two methods.

**Table 5:** The method of sampling the posterior distribution of the MCMC chain by averaging random accepted models from the steady state was compared to the method of selecting the model with the overall maximum log likelihood.

				**Order 3 model**			**Order 4 model**		
				
**Org 1**	**Org 2**	**Frag L**	**Sampling type**	***D*_3_**	**Accuracy**	**LL**	***D*_4_**	**Accuracy**	**LL**
*Arthrobacter aurescens TC1 *vs. *Sinorhizobium meliloti 1021*									

		400	Steady state sampled	1.08	0.95	-1054490.36			
							1.09	0.94	-1040007.41
		400	Maximum log likelihood	1.02	0.94	-1055584.16			
NC_003047	NC_008711								
		1000	Steady state sampled	1.95	0.97	-2648159.80			
							2.52	0.99	-2637429.69
		1000	Maximum log likelihood	2.12	0.98	-2645204.57			

*Lactococcus lactis subsp. cremoris MG1363 *vs. *Francisella tularensis subsp. holarctica FTA*									

		400	Steady state sampled	1.08	0.90	-1045063.72			
							1.33	0.95	-1040811.10
		400	Maximum log likelihood	1.15	0.92	-1047966.99			
NC_009004	NC_009749								
		1000	Steady state sampled	2.02	0.96	-2624742.76			
							2.22	0.97	-2615376.71
		1000	Maximum log likelihood	2.19	0.96	-2626080.18			

*Helicobacter pylori HPAG1 *vs. *Streptococcus pneumoniae R6*									

		400	Steady state sampled	0.93	0.96	-1059955.55			
							1.18	0.93	-1045561.25
		400	Maximum log likelihood	0.97	0.96	-1061298.85			
NC_003098	NC_008086								
		1000	Steady state sampled	1.71	0.99	-2656860.50			
							2.28	0.99	-2634722.55
		1000	Maximum log likelihood	1.69	0.98	-2658488.27			

*Staphylococcus aureus RF122 *vs. *Prochlorococcus marinus str. NATL2A*									

		400	Steady state sampled	0.99	0.90	-1049716.33			
							1.00	0.95	-1045188.54
		400	Maximum log likelihood	0.99	0.93	-1050316.80			
NC_007335	NC_007622								
		1000	Steady state sampled	1.92	0.97	-2636903.64			
							2.21	0.97	-2624299.41
		1000	Maximum log likelihood	1.75	0.97	-2636046.52			

*Staphylococcus aureus subsp. aureus COL *vs. *Methanocaldococcus jannaschii DSM 2661*									

		400	Steady state sampled	0.96	0.95	-1037936.55			
							1.05	0.89	-1033285.36
		400	Maximum log likelihood	0.92	0.94	-1037505.67			
NC_000909	NC_002951								
		1000	Steady state sampled	1.84	0.98				
							2.36	0.99	-2581181.80
		1000	Maximum log likelihood	1.94	0.98	-2601394.32			

Frag L, Fragment length; LL, Output model log likelihood

The resulting accuracy differences were negligible. Accuracy was also compared in 3-mer models vs. 4-mer models. While 4-mer models slightly outperformed 3-mer models on average, a significant run time increase was observed (not shown). NC_identifiers refer to GenBank accession numbers for genomes listed in each trial.

The algorithm is implemented in portable Perl and C code that can be compiled and run on any platform supporting a Perl interpreter. Both memory use and run time scale linearly with the number of fragments and species, and sub-linearly with fragment length. Memory complexity scales quadratically with the number of dimensions in the search space, or exponentially with *k *(as shown in Table 1). We selected *k *= 3 as the default *k*-mer length, with user-defined options for 2, 4, or 5 available. We have not yet formalized convergence time performance as a function of *k*. In practice, a 3-species dataset of 1000 fragments per species, with *k*-mer order set to 3, takes approximately 2 minutes to run on an Intel Core 2 Duo-class processor.

## Conclusion

We developed an unsupervised, maximum likelihood approach to the binning problem - called LikelyBin. LikelyBin uses a MCMC framework to estimate the set of master distributions and relative frequencies most likely to give rise to an observed collection of short reads. The likelihood approach is based on *k*-mer distributions, for which we developed an index of separability of any pair of genomes, which we termed the genomic fragment divergence measure, *D*_*n*_. We found that the vast majority of genomes have sufficient divergence to be distinguished using the present method (Figure 3).

Using a high-performance implementation, LikelyBin can be used to cluster sequences with high accuracy (in some cases, > 95%) even when the mononucleotide content of the original genomes is essentially identical (Figure 4). The method does as well or better than a comparable semi-supervised method (CompostBin [25]) that also uses *k*-mer distributions as the statistical basis for binning (Table 5).

Performance of LikelyBin is consistently good for synthesized low-complexity datasets (2-10 species) with fragments of length as low as 400 nt, which corresponds to the characteristic single-read length of a 454 pyrosequencing FLX machine. Microread sequencing technologies such as Solexa and SOLiD are currently out of reach of any non-alignment-based binning method when applied to single reads, which range from 30 to 50 base pairs with these technologies.

The unsupervised nature of our approach makes it potentially useful for classifying mixtures of novel sequences for which supervised learning-based methods may have difficulties. A future direction for our work is to combine our statistical formalism with alignment and supervised composition-based models. For example, we could develop a feature selection framework that would transform the input fragments' features such as *k*-mer statistics, coding frame information, and variable-length motifs into a lower-dimensional space. We could then feed these features to an unsupervised MCMC-based classifier in tandem with an alignment-based classifier that can partially label fragments based on known taxonomic information, then compare and combine their results.

A number of challenges remain to broaden the scope and applicability of the current method. At present, our method is scalable for *k*-mer length from *k *= 2 to *k *= 5. We intend to expand the method's ability to capture longer motif frequencies by using dimension transformation or feature selection in a future work. Intra-genomic heterogeneity of oligonucleotide distributions is another topic that is yet to be addressed. A confidence measure that serves as a performance self-check is already available as part of our method but we have not incorporated it into the program's output yet.

Further, applying the current method in an environmental context requires an estimation of the number of bins. The problem of identifying the necessary number of distinct models, or groups thereof, to represent all components of a given genome, is related to the problem of identifying the number of distinct genomes in the mixture. A combination of jump diffusion and grouped models is our currently planned solution. In this respect, the use of phylogenetic markers to estimate the number of bins will provide important prior information.

In summary, the unsupervised method we proposed is based on a maximum likelihood formalism and can bin short fragments (*L *= 400 nt) of low complexity communities (2-10 species) with high accuracy (in some cases, > 95%) given sufficient genomic divergence. The maximum likelihood formalism and its MCMC implementation make the current approach amenable to extension and incorporation into other packages. The MCMC binner application is provided as an open-source downloadable package, LikelyBin [33], that can be installed on any platform that supports Perl and C and is fully automated to facilitiate use in genome processing pipelines. Version 0.1 of the source code is provided in Additional files 4.

## Authors' contributions

AK developed code, performed all statistical analyses, and wrote the manuscript. SB developed code and performed preliminary statistical analyses. JD developed the mathematical method and supervised the statistical analysis. JSW developed the mathematical method, supervised the computational and statistical analysis, and wrote the manuscript. All authors read and approved the final manuscript.

## Appendix

### Example application of likelihood model

Suppose we have two source genomes, *G*_1 _and *G*_2_, with two fragments from each: *G*_1 _→ {ATGTTA, TGTAAT}, *G*_2 _→ {CCTGTC, AGGCCTC}. We wish to evaluate the likelihood of observing these sequences according to a dimer model of 2 sources, *M *= {*S*_1_, *S*_2_}, which we have generated. Assume the model's source frequency vector is *F *= [0.6, 0.4], its monomer frequencies are

{*S*_1 _: {*p*_*A *_= 0.3, *p*_*T *_= 0.3, *p*_*G *_= 0.2, *p*_*C *_= 0.2}, *S*_2 _: {*p*_*A *_= 0.2, *p*_*T *_= 0.2, *p*_*G *_= 0.3, *p*_*C *_= 0.3}} and its dimer frequencies are

*S*_1 _: {*p*_*AA *_= 0.09, *p*_*AT *_= 0.09, *p*_*AG *_= 0.06, *p*_*AC *_= 0.06, *p*_*TA *_= 0.07, *p*_*TT *_= 0.09, *p*_*TG *_= 0.06, *p*_*TC *_= 0.08 *p*_*GA *_= 0.08, *p*_*GT *_= 0.06, *p*_*GG *_= 0.04, *p*_*GC *_= 0.02*p*_*CA *_= 0.06, *p*_*CT *_= 0.06, *p*_*CG *_= 0.04, *p*_*CC *_= 0.04}, *S*_2 _: {*p*_*AA *_= 0.02, *p*_*AT *_= 0.04, *p*_*AG *_= 0.08, *p*_*AC *_= 0.06, *p*_*TA *_= 0.04, *p*_*TT *_= 0.02, *p*_*TG *_= 0.06, *p*_*TC *_= 0.08, *p*_*GA *_= 0.08, *p*_*GT *_= 0.06, *p*_*GG *_= 0.07, *p*_*GC *_= 0.09*p*_*CA *_= 0.06, *p*_*CT *_= 0.08, *p*_*CG *_= 0.09, *p*_*CC *_= 0.07}}

Then the likelihoods of observing the first fragment, ATGTTA, given master distributions *S*_1 _and *S*_2_, respectively, are



where superscripts *S*_1 _and *S*_2 _denote the master distribution. Similarly,



The overall posterior likelihood of the model is then



## Supplementary Material

Additional file 1 **Convergence dynamics**. Figure 1: Convergence dynamics for good accuracy, *Mycoplasma capricolum subsp. capricolum ATCC 27343 *vs. *Campylobacter jejuni subsp. jejuni 81-176 *(*D*_3 _= 2.8). A single MCMC simulation was completed for this pair of genomes as described in Methods. *k*-mer order 3 model was used with 30000 steps, and expected nucleotide frequencies in accepted models were plotted over time for all independent mono- and dinucleotides in the model. Two starting conditions were compared: uniform initial frequencies (solid line) and frequencies at dataset mean (dashed line). Dotted lines indicate true average frequencies in the constituent species' fragment datasets. Convergence was observed to be substantially the same, demonstrating robustness of the algorithm to initial starting conditions. Final model accuracy was ≈ 95% in both cases. Click here for file

Additional file 2 **Convergence dynamics**. Figure 2: Convergence dynamics for poor accuracy, *Granulibacter bethesdensis CGDNIH1 *vs. *Gluconobacter oxydans 621H *(*D*_3 _= 0.45). Details are identical to Additional file 1, but final model accuracy was ≈ 60% in both cases. Click here for file

Additional file 3 **Accuracy-divergence dependencies for Bayesian sampling**. Figure 3: Pairs and triples of genomes were sampled randomly from a set of 1055 completed bacterial chromosomes, and experiments were conducted using Bayesian posterior distribution sampling on the stationary distribution of the MCMC simulation. The results were found to not be significantly different from those for maximum likelihood sampling (Figure 4). Click here for file

Additional file 4 **LikelyBin version 0.1 archive**. This archive contains the source and executable files for the binner application. Click here for file
