# Supplementary figures and images for: Unsupervised statistical clustering of environmental shotgun sequences
Source: BMC Bioinformatics. 2009 Oct 2;10:316. doi: 10.1186/1471-2105-10-316 (PMC2765972; doi:10.1186/1471-2105-10-316)

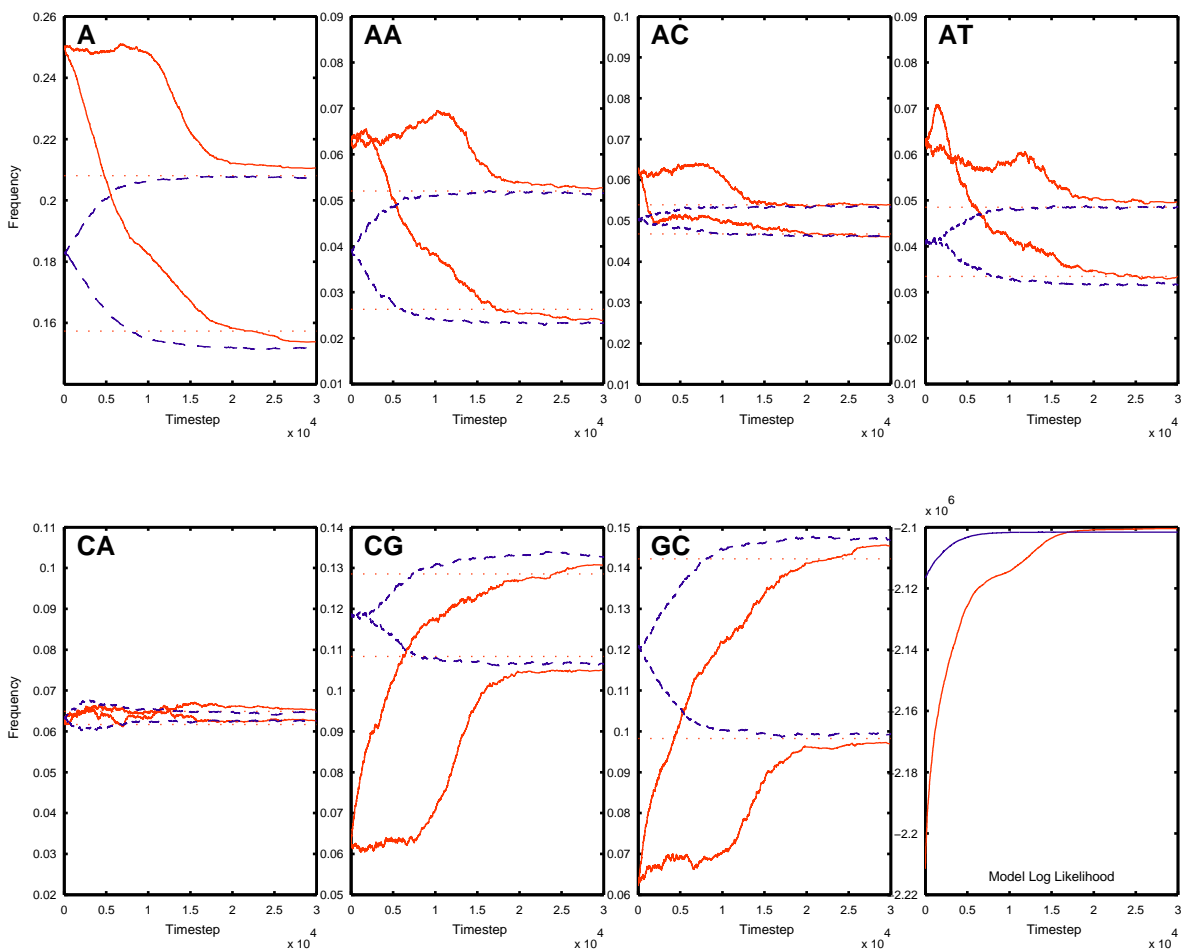

Supplement: Additional file 1 — Convergence dynamics. Figure 1: Convergence dynamics for good accuracy, Mycoplasma capricolum subsp. capricolum ATCC 27343 vs. Campylobacter jejuni subsp. jejuni 81-176 (D3 = 2.8). A single MCMC simulation was completed for this pair of genomes as described in Methods. k-mer order 3 model was used with 30000 steps, and expected nucleotide frequencies in accepted models were plotted over time for all independent mono- and dinucleotides in the model. Two starting conditions were compared: uniform initial frequencies (solid line) and frequencies at dataset mean (dashed line). Dotted lines indicate true average frequencies in the constituent species' fragment datasets. Convergence was observed to be substantially the same, demonstrating robustness of the algorithm to initial starting conditions. Final model accuracy was ≈ 95% in both cases. [file 1471-2105-10-316-S1.PDF]

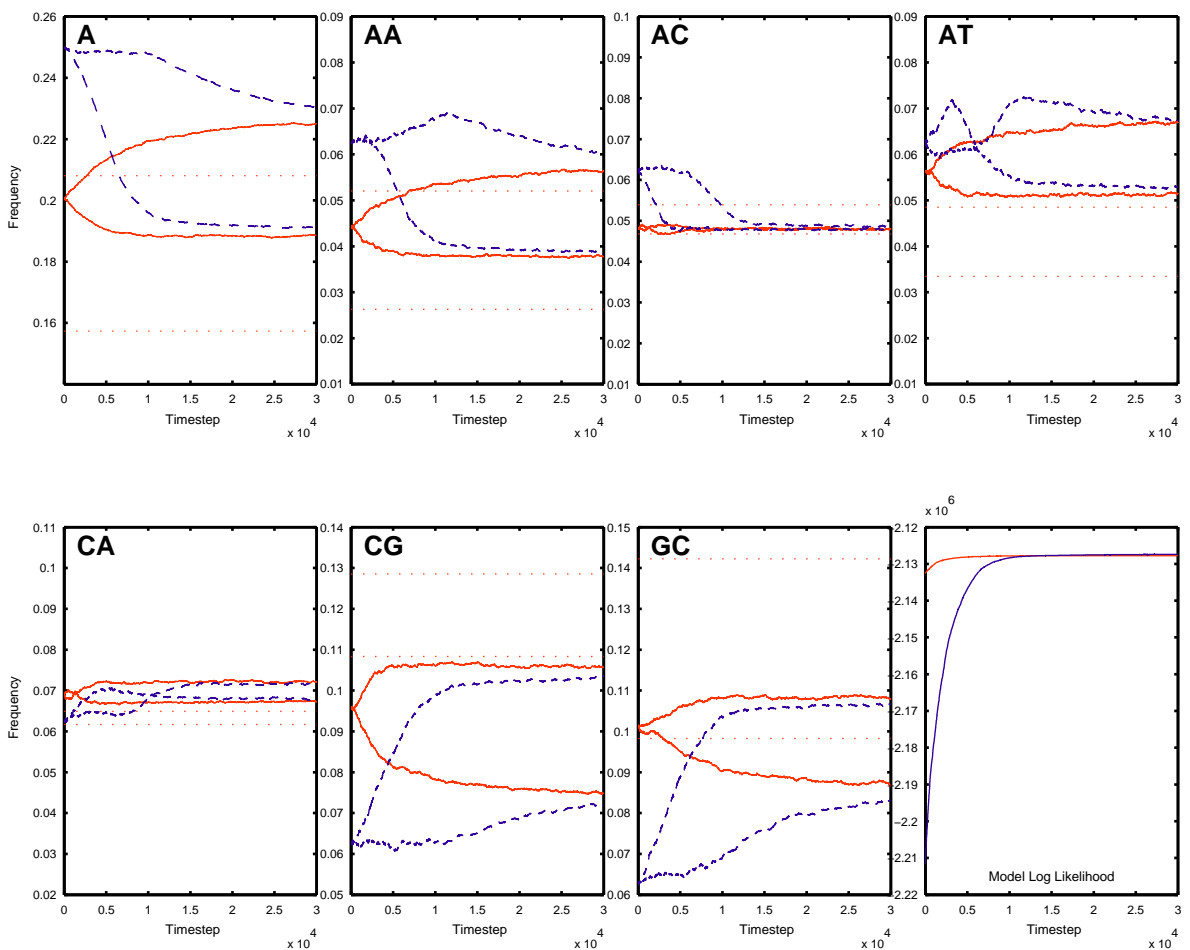

Supplement: Additional file 2 — Convergence dynamics. Figure 2: Convergence dynamics for poor accuracy, Granulibacter bethesdensis CGDNIH1 vs. Gluconobacter oxydans 621H (D3 = 0.45). Details are identical to Additional file 1, but final model accuracy was ≈ 60% in both cases. [file 1471-2105-10-316-S2.PDF]

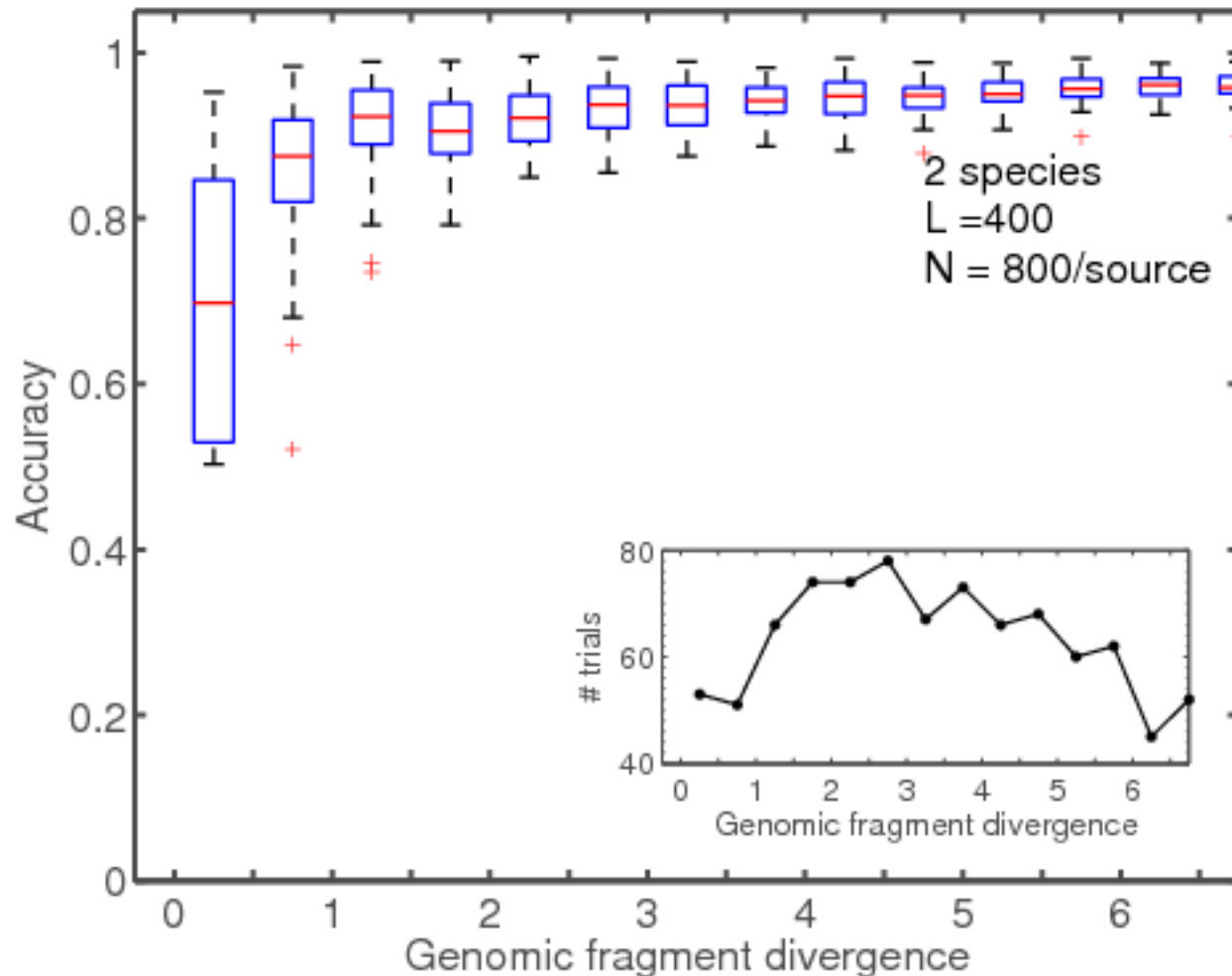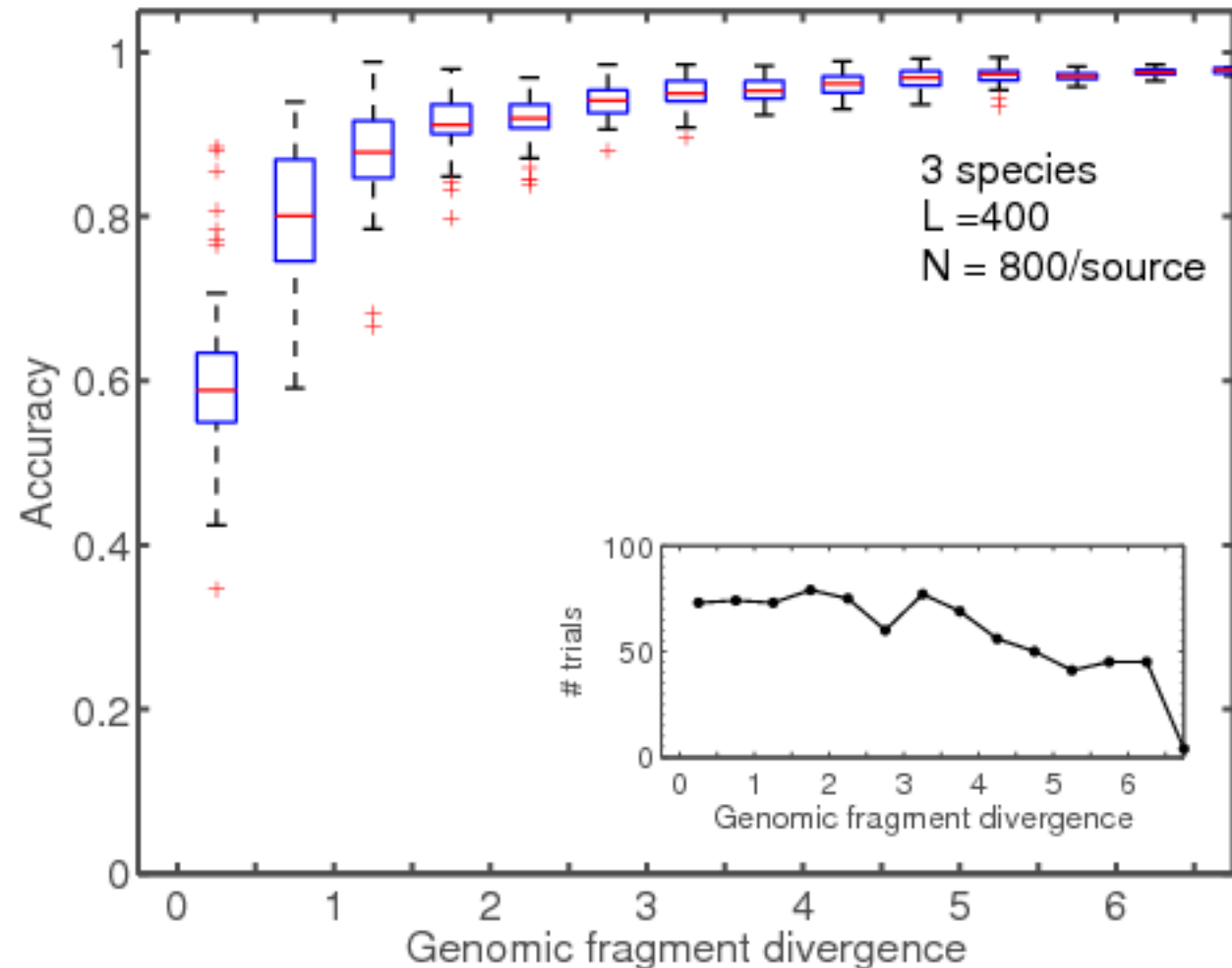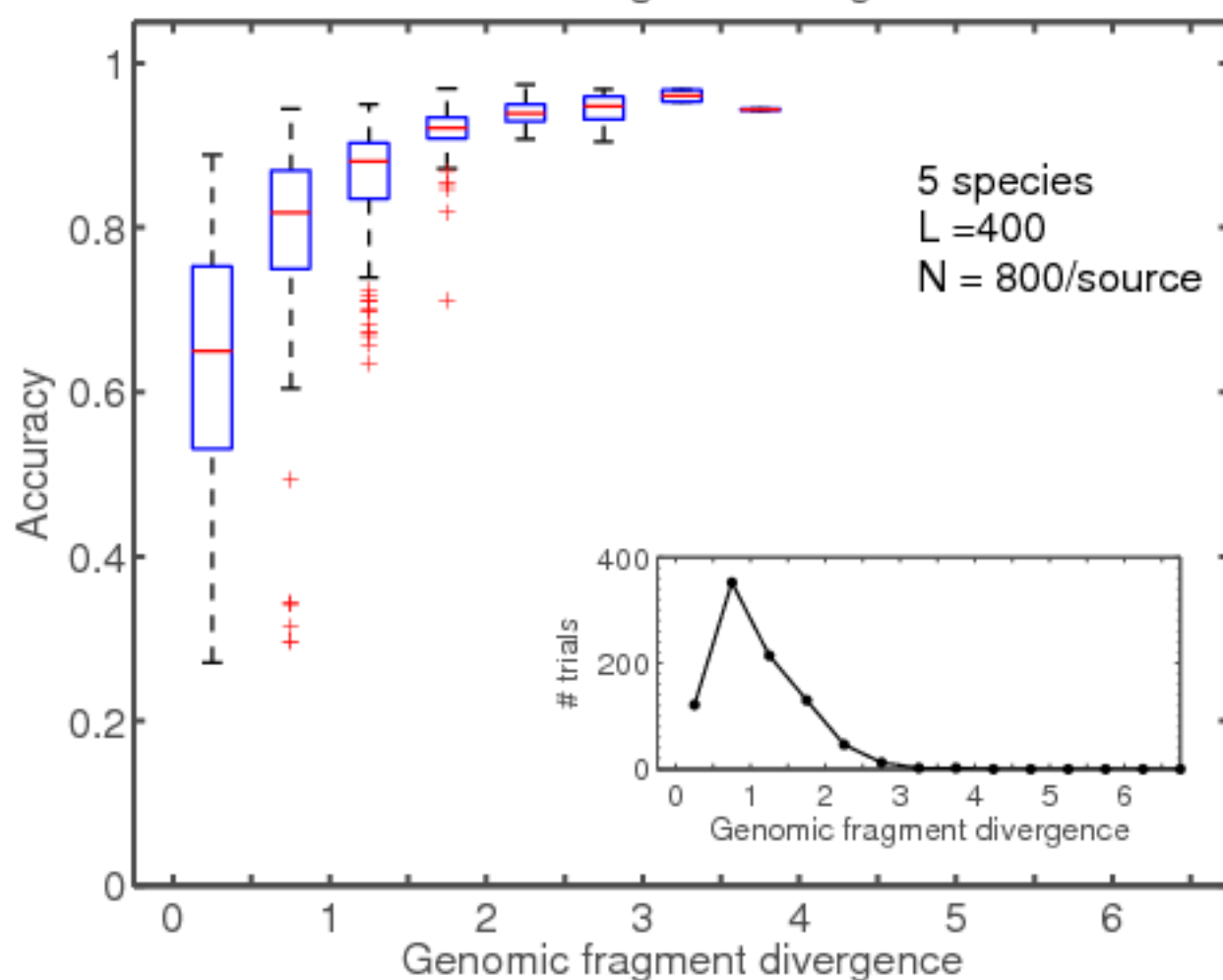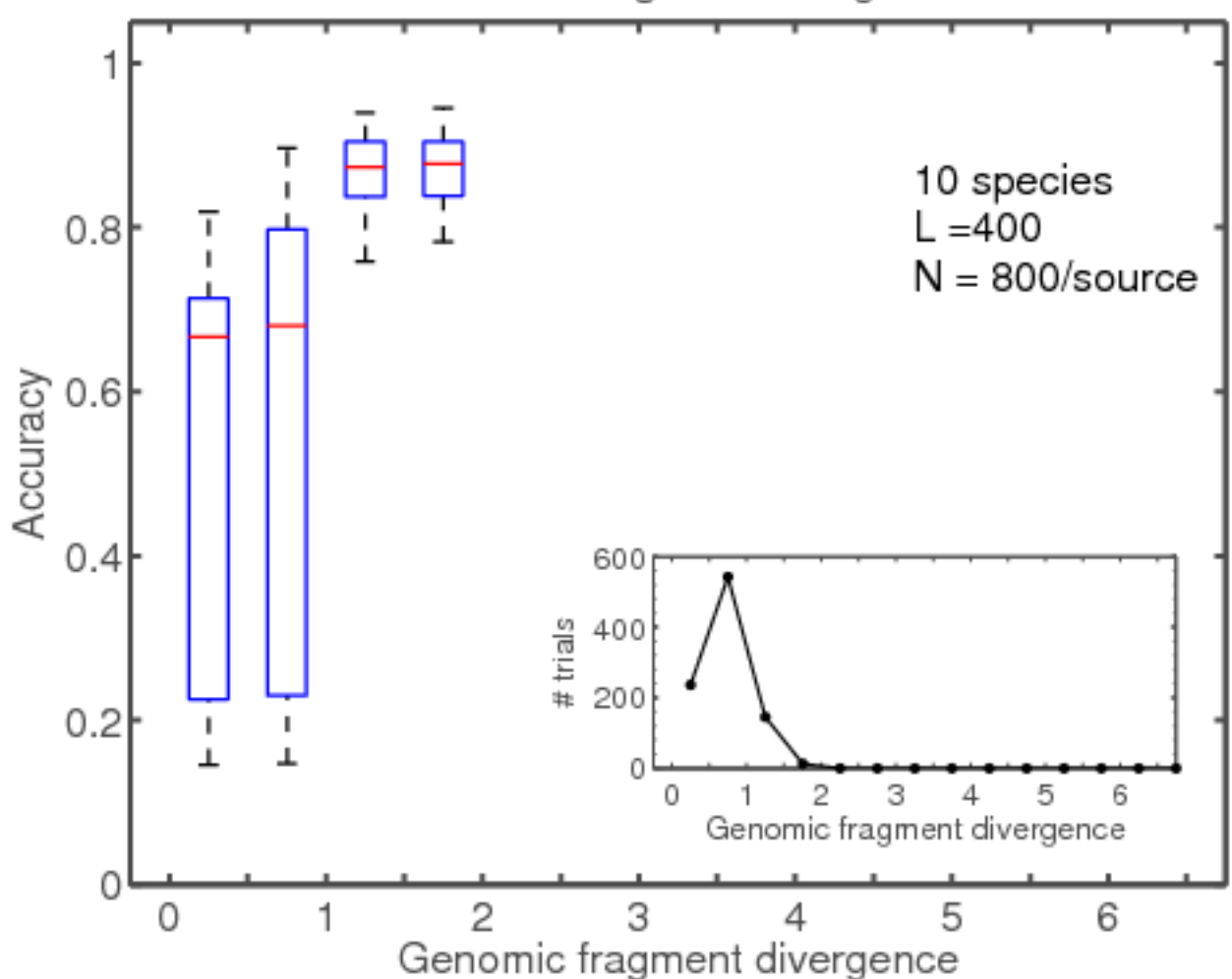

Supplement: Additional file 3 — Accuracy-divergence dependencies for Bayesian sampling. Figure 3: Pairs and triples of genomes were sampled randomly from a set of 1055 completed bacterial chromosomes, and experiments were conducted using Bayesian posterior distribution sampling on the stationary distribution of the MCMC simulation. The results were found to not be significantly different from those for maximum likelihood sampling (Figure 4). [file 1471-2105-10-316-S3.PDF]
